# Insecticide resistance in malaria and arbovirus vectors in Papua New Guinea, 2017–2022

**DOI:** 10.1186/s13071-022-05493-3

**Published:** 2022-11-14

**Authors:** Michelle Katusele, Solomon Lagur, Nancy Endersby-Harshman, Samuel Demok, Joelyn Goi, Naomi Vincent, Muker Sakur, Absalom Dau, Lemen Kilepak, Stephen Gideon, Christine Pombreaw, Leo Makita, Ary Hoffmann, Leanne J. Robinson, Moses Laman, Stephan Karl

**Affiliations:** 1 PNG Institute of Medical Research, Madang, Madang Province Papua New Guinea; 2 School of BioSciences, Bio21 Institute, The University of Melbourne, Parkville, Victoria Australia; 3 Burnet Institute of Medical Research, Melbourne, Victoria Australia; 4 Papua New Guinea National Department of Health, Port Moresby, National Capitol District Papua New Guinea; 5 School of Public Health and Preventive Medicine, Monash University, Melbourne, Victoria Australia; 6 Australian Institute of Tropical Health and Medicine, James Cook University, Smithfield, Queensland Australia

**Keywords:** Mosquitoes, Insecticide resistance, Malaria, Arbovirus, Vector control, Public health

## Abstract

**Background:**

Insecticide resistance (IR) monitoring is essential for evidence-based control of mosquito-borne diseases. While widespread pyrethroid resistance in *Anopheles* and *Aedes* species has been described in many countries, data for Papua New Guinea (PNG) are limited. Available data indicate that the local *Anopheles* populations in PNG remain pyrethroid-susceptible, making regular IR monitoring even more important. In addition, *Aedes aegypti* pyrethroid resistance has been described in PNG. Here, *Anopheles* and *Aedes* IR monitoring data generated from across PNG between 2017 and 2022 are presented.

**Methods:**

Mosquito larvae were collected in larval habitat surveys and through ovitraps. Mosquitoes were reared to adults and tested using standard WHO susceptibility bioassays. DNA from a subset of *Aedes* mosquitoes was sequenced to analyse the voltage-sensitive sodium channel (*Vssc*) region for any resistance-related mutations.

**Results:**

Approximately 20,000 adult female mosquitoes from nine PNG provinces were tested. *Anopheles punctulatus *sensu lato mosquitoes were susceptible to pyrethroids but there were signs of reduced mortality in some areas. Some *Anopheles* populations were also resistant to DDT. Tests also showed that *Aedes. aegypti* in PNG are resistant to pyrethroids and DDT and that there was also likelihood of bendiocarb resistance. A range of *Vssc* resistance mutations were identified. *Aedes* *albopictus* were DDT resistant and were likely developing pyrethroid resistance, given a low frequency of *Vssc* mutations was observed.

**Conclusions:**

*Aedes aegypti* is highly pyrethroid resistant and also shows signs of resistance against carbamates in PNG. *Anopheles punctulatus *s.l. and *Ae. albopictus* populations exhibit low levels of resistance against pyrethroids and DDT in some areas. Pyrethroid-only bed nets are currently the only programmatic vector control tool used in PNG. It is important to continue to monitor IR in PNG and develop proactive insecticide resistance management strategies in primary disease vectors to retain pyrethroid susceptibility especially in the malaria vectors for as long as possible.

**Graphic abstract:**

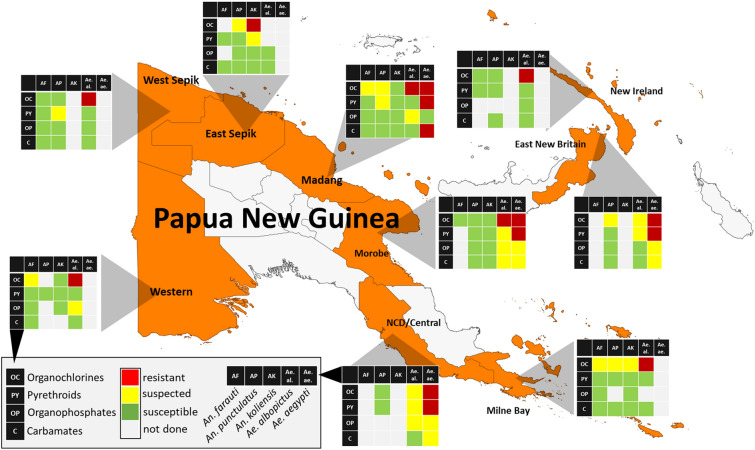

## Background

Insecticide resistance (IR) monitoring in mosquitoes is important for ensuring continued efficacy of insecticide-based vector control interventions and for guiding the selection and application of the most appropriate combinations of products and active ingredients (AIs) [1, 2]. This includes the choice of products for long-lasting insecticidal nets (LLINs), indoor residual spraying and other vector control tools. Ideally, a combination of products with complementary AIs is used, and mosquito populations should be fully susceptible against at least one of the AIs in the combination [3].

Papua New Guinea (PNG) is endemic for malaria and lymphatic filariasis, both of which are transmitted by anopheline mosquitoes [4, 5], and a wide range *of Aedes*-transmitted arboviruses, including dengue and chikungunya viruses [6, 7]. The primary *Anopheles* mosquito vector species in PNG belong to the *Anopheles punctulatus* complex including *An. farauti *sensu stricto, *An. koliensis* and *An. punctulatus* s.s. Secondary species that have been demonstrated to transmit malaria include *An. longirostris* complex and *An. bancroftii* [8, 9]. Arbovirus vectors including *Aedes aegypti* are found in larger, more densely populated areas, whereas *Ae. albopictus* dominates in rural settings and smaller urban centres [10]. Locally important secondary *Aedes* species include *Ae. scutellaris* [11, 12]*.*

Over recent years we have reported IR status of malaria [13, 14] and arbovirus [15] vectors in several locations in PNG, determined using World Health Organization (WHO) insecticide susceptibility bioassays. Before this study, data on IR were limited to five provinces for *Anopheles* spp. and two provinces for *Aedes* spp. Full susceptibility of all tested anopheline mosquitoes against pyrethroids (deltamethrin), carbamates (bendiocarb) and organophosphates (malathion) and potential resistance to organochlorides [dichlorodiphenyltrichloroethane (DDT)] was reported [13, 14]. Furthermore, pyrethroid resistance in *Ae. aegypti* and DDT resistance in *Ae. albopictus* were observed in Port Moresby and Madang [15]. Genetic analysis indicated that all *Ae. aegypti* in PNG carried common target site resistance mutations conferring resistance to pyrethroids and DDT [15], though no genetic mutations conferring DDT resistance in *Ae. albopictus* and *Anopheles* mosquitoes were previously identified. Further studies are required to better understand the mechanisms underlying the observed DDT resistance in these mosquito species.

This study provides updated data of IR monitoring in PNG that includes bioassay data from nine PNG provinces for *Anopheles* and *Aedes* mosquitoes conducted between 2017 and 2022. This update provides a comprehensive overview of the distribution and current IR status in PNG disease vector populations. Our studies focus on the low altitude areas of PNG where vector-borne disease transmission is intense [6, 16].

## Methods

### Study site and mosquito sampling

**Fig. 1:**
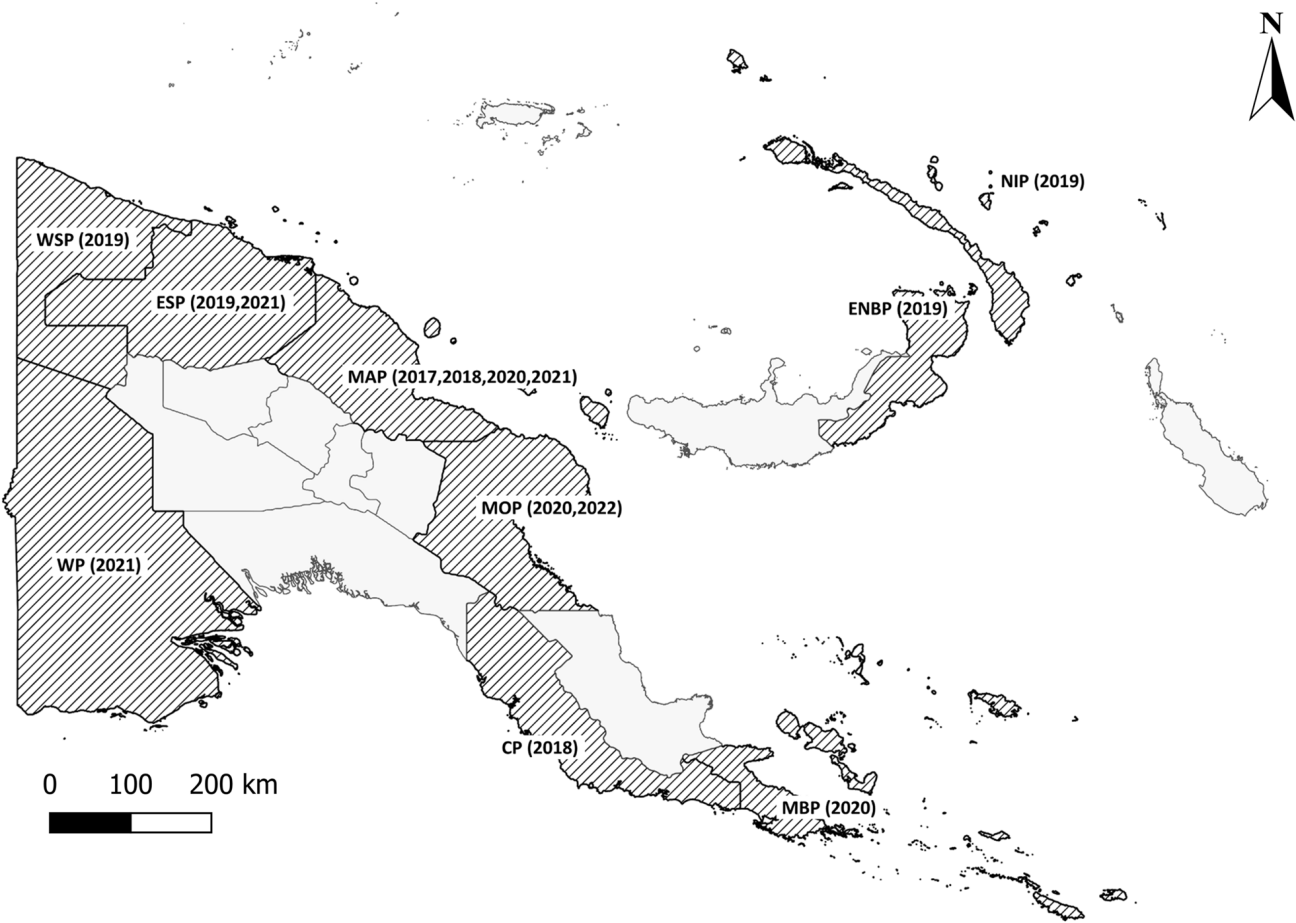
Map of Papua New Guinea and survey provinces. The provinces in which surveys were carried out are shaded and abbreviated as follows: National Capital District, Central Province (CP), Milne Bay Province (MBP), Western Province (WP), East New Britain (ENBP), New Ireland (NIP), East Sepik (ESP), Madang (MAP), Morobe (MOP) and West Sepik (WSP). The years when surveys were completed are shown in parentheses

The study was conducted across nine provinces of PNG (Fig. 1), with high burdens of vector-borne diseases between 2017 and 2022. The provinces included National Capital District (NCD), Central Province, Milne Bay Province and Western Province in the southern region of mainland PNG; East New Britain and New Ireland in the New Guinea Islands region and East Sepik, Madang, Morobe and West Sepik on the northern coast of mainland PNG. Most provinces were surveyed once, East Sepik Province was surveyed twice, and Madang Province was surveyed four times.

Provinces were surveyed for anopheline and aedine mosquito larvae simultaneously. *Anopheles* larval habitats included drainage channels, sand-barred streams, transient puddles, forest swamps, pig-wallows, wells and riverine puddles. The collection of larvae in each province was limited by access via the road network and collections were conducted mainly along the sides of roads accessible by vehicle. On average, each survey included 14 days of larval collections. *Anopheles* larvae were collected, preferably as third or fourth instars, from their habitats using plastic scoops, placed into 500-ml plastic bottles, labelled and brought back to a field insectary or to the PNG Institute of Medical Research (PNGIMR) insectary in Madang. Larval habitat characteristics of each sampled larval source were recorded, including Global Positioning System (GPS) locations. Data were entered into the electronic data capture system Epicollect5 (https://five.epicollect.net/; V4.2.0 Centre for Genomic Pathogen Surveillance, 2022) using mobile electronic tablets. For reference, bioassays were also conducted with a fully susceptible *An. farauti* colony maintained at PNGIMR. The colony was originally from Rabaul, ENB, and established at the PNGIMR in 2008. It has been routinely tested with susceptibility confirmed in various studies since 2009 [13, 14, 17, 18].

*Aedes* eggs and larvae were collected in ovitraps [19, 20] and through larval habitat sampling. A total of around 20 ovitraps per survey were set up in consenting households in an urban area (town or suburb) for 5 days. A single trap was placed in a protected location outside each house and the GPS location was logged electronically in Epicollect5. Additionally, common aedine mosquito larval habitats were sampled and habitat characteristics recorded as described for anopheline mosquitoes.

### Mosquito rearing

Temporary insectaries were set up for each survey except in Madang province, where the PNGIMR has a permanent insectary. Mosquito larvae (both *Anopheles* spp. and *Aedes* spp.) were reared in shallow photo-developing trays (sizes 16.5 × 11 cm or 24.5 × 18.5 cm or depending on the number of larvae collected per site, with larval density ranging from 200 to 500 per respective tray size). The larvae were fed with ground Marine Master Tropical fish flakes (Marinepet Australia Pty Ltd). Pupae were collected daily into small plastic cups, and cups were placed into screened plastic containers (20 cm in diameter and 20 cm in height) or BugDorm-1 insect rearing cages (MegaView Science Co., Ltd, Taiwan), with capacity to hold 300–500 adult mosquitoes at a time. Emerged adult mosquitoes were kept for 3–5 days in the cages before being used in WHO tube bioassays, with access to cotton balls soaked with 10% (w/v) sucrose solution placed on top of the cages and covered with damp cloth to keep ambient tropical temperature and humidity levels.

### WHO insecticide susceptibility bioassays

*Anopheles *(mostly *An. punctulatus *s.l.) and *Aedes *(mostly *Ae. aegypti* and *Ae. albopictus*) mosquitoes were tested against eight insecticides. *Anopheles* mosquitoes were tested against WHO recommended discriminating concentrations [2, 21] of the following: pyrethroid type I insecticide, 0.75% permethrin; pyrethroid type II insecticides, 0.05% deltamethrin, 0.05% lambda-cyhalothrin and 0.05% alphacypermethrin; carbamate insecticide, 0.1% bendiocarb; organochloride insecticide, 4% DDT; and organophosphate insecticides, 5% malathion and, 0.25% pirimiphos-methyl. These concentrations were also used to test *Aedes* mosquitoes. We also included the discriminating concentrations of 0.03% deltamethrin and 0.8% malathion against *Aedes* populations [21, 22] in Morobe and NCD. The number of insecticides tested varied across surveys (subject to availability of mosquito larvae). Insecticides such as deltamethrin and DDT were prioritized because of their current (deltamethrin in LLINs) and historical (DDT for IRS) usage in the country.

Bioassays were conducted using WHO standard procedures [2], with 25 adult female anopheline mosquitoes per bioassay tube, and a full test having four replicates and two control tubes (i.e., *n* = 150 adult female mosquitoes per full test). However, if insufficient larvae were present to conduct a full test, tests were conducted with 20 to 90 mosquitoes (one to three replicates and at least one negative control per test). Mosquitoes were exposed to insecticide-impregnated filter papers inside the replicate tubes for a total of 60 min, with knockdown recorded at 5 min intervals for 30 min, followed by 10 min interval readings until 60 min. Mosquitoes were then transferred from each tube into separate holding tubes (controls included) and kept at ambient tropical temperature and humidity for a 24 h holding period, with access to 10% (w/v) sucrose solution. The primary endpoint was 24 h mortality for all insecticides tested; however, we also included a 48-h holding period and mortality observation for DDT, as we observed much slower rates of knockdown with this insecticide. Mosquitoes were graded as recommended by WHO [2]; alive mosquitoes were able to stand and flew in a coordinated manner whereas moribund mosquitoes were knocked down and or could not fly in a coordinated manner or fell down immediately after taking flight; dead mosquitoes showed no signs of life and could not stand. Mortality rates were calculated by dividing the sum of all dead and moribund mosquitoes by the total number of mosquitoes tested and multiplied by 100 [2].

Mortality from each holding tube was recorded after 24 h, with all survivors separated for identification during later laboratory analysis. All mosquitoes that were alive after the 24 h holding period (test survivors and controls) were anesthetized at −20 °C and morphologically identified using standard identification keys [23] along with the rest of the tested mosquitoes.

### Molecular analysis

Genomic DNA analysis was conducted at the Bio21 Institute in Australia. Genomic DNA was extracted from a sample of adult mosquitoes (55 *Ae*. *aegypti* and 170 *Ae*. *albopictus*) using the Roche High Pure PCR template kit (Roche Molecular Systems, Inc.) according to the instructions of the manufacturer, but with two elution steps (first elution in 60 µl elution buffer and second elution in 120 µl). The second elution was diluted 1:10 with water and used in the molecular analysis of resistance mutations.

Custom TaqMan^®^ SNP Genotyping Assays (Life Technologies), developed for each of the three target site mutations in the voltage-sensitive sodium channel (*Vssc*) of *Ae. aegypti* (codons 989, 1016, 1534), were run on the Roche LightCycler^®^ 480 and analysed using the endpoint genotyping method [24]. The *Vssc* amino acid positions are labelled as S989P, V1016G and F1534C according to the sequence of the most abundant splice variant of the house fly, *Musca domestica*, *Vssc* (GenBank accession nos. AAB47604 and AAB47605 [25]).

The *Vssc* codon 1534 of *Ae*. *albopictus* was screened for mutations using PCR primers aegSCF7/aegSCR7 and sequenced with aegSCR8 [26]; 2 µl genomic DNA were amplified in a 25-µl PCR mix that included final concentrations of ThermoPol buffer Mg-free (1×) (New England Biolabs, Ipswich MA, USA), dNTPs (200 µM each), MgCl_2_ (1.5 mM), 0.5 µM each of forward and reverse primers, 0.625 units of Immolase^™^ Taq polymerase (Bioline, London, UK) and PCR-grade H_2_O. Thermocycling conditions followed those of Ahmad et al. [27]. PCR amplicons (740 bp) were sent to Macrogen Inc. (Seoul, South Korea) for Sanger sequencing on a 3730 × l DNA analyser. Sequences were analysed, aligned and trimmed (~ 180 bp) using the programme Geneious Prime^®^ 2020.0.4 (Biomatters Ltd.).

### Data analysis

Mosquito populations were classified as resistant or susceptible according to the WHO criteria [2] as follows: mortality rate of 98–100% is susceptible, 90–97% suggests possible resistance and < 90% indicates confirmed resistance. Proportion of knocked down or dead mosquitoes was calculated and 24 h mortality was adjusted using ‘Abbott’s formula’ when the control mortality was between 5 to 20%. Analysis of proportions (95% CI) and one-sample *z*-tests using the online calculator Epitools (https://epitools.ausvet.com.au/; AusVet, 2022) were used to determine whether the observed 24 h mortality was statistically indicative of resistance or susceptibility. Analyses were conducted for each species (where possible), insecticide and province.

## Results

### Species distribution of mosquitoes tested in the bioassays

**Fig. 2:**
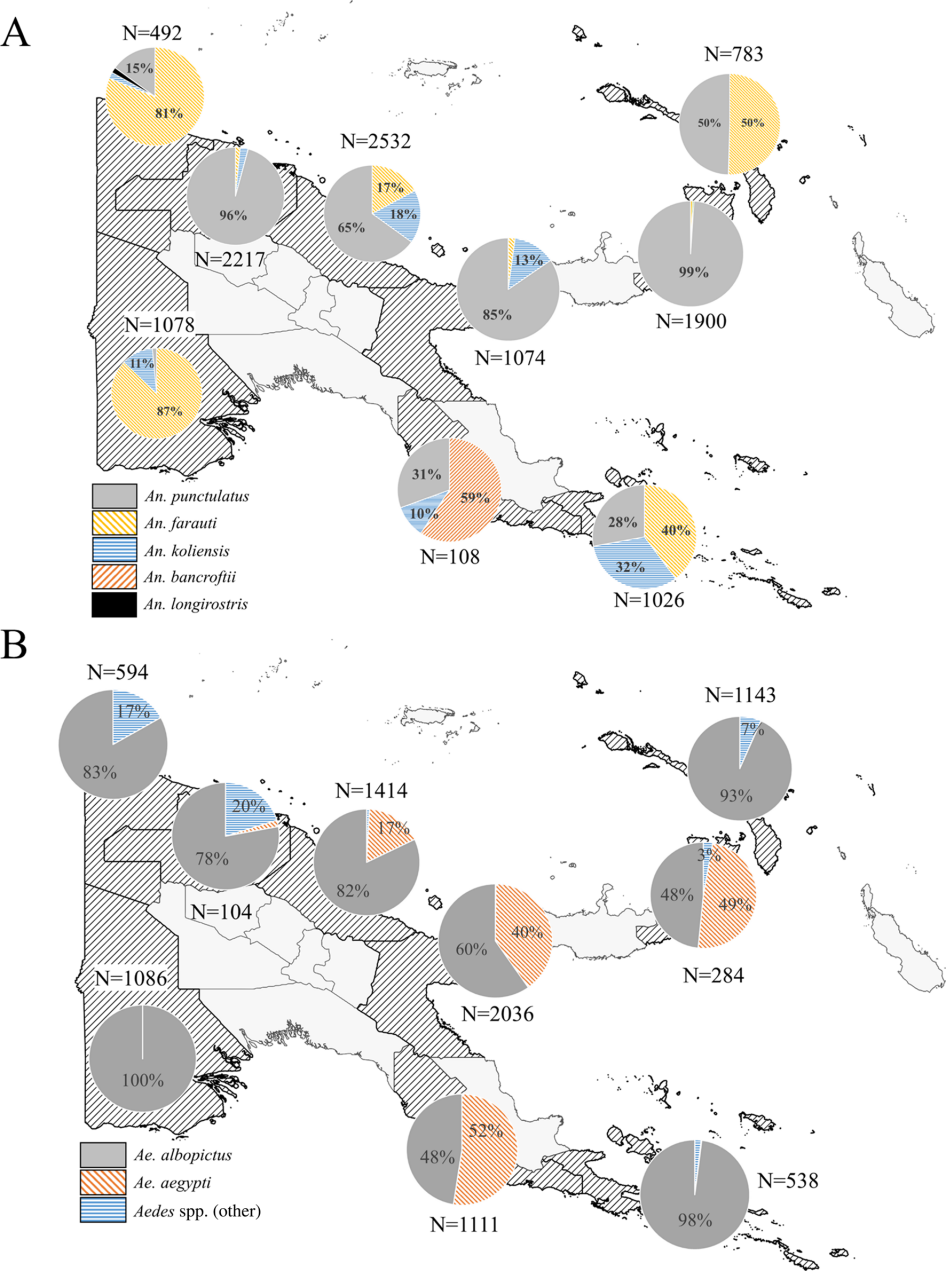
Mosquito species distribution used in WHO tube bioassays. **A**: *Anopheles* species; **B**: *Aedes* species. Percentages < 2% are not explicitly presented in the pie charts. The total number of mosquitoes exposed in the bioassays per province is also shown (N)

A total of 11,210 *Anopheles* mosquitoes and 8294 *Aedes* mosquitoes were used in the WHO tube bioassays across nine provinces in PNG between 2017 and 2022. The distribution of taxa identified based on morphology is shown in Fig. 2. Bioassays were also conducted with a pyrethroid susceptible *An. farauti* colony at PNGIMR Madang (*n* = 1762).

### Overall insecticide resistance profile of PNG vector species

Table 1 shows the overall insecticide susceptibility profiles of *An. farauti* colony mosquitoes, *An. punctulatus* s.l. *Ae. albopictus*, *Ae. aegypti* and secondary species (combined) as determined in the present study.

**Table 1:** Summary of phenotypic resistance bioassay results of the primary malaria and arbovirus vector species in PNG

Insecticide	*An. farauti* s.s.^a^		*An. punctulatus* s. l.		*Anopheles*. spp.		*Ae. albopictus*		*Ae. aegypti*		*Aedes.* spp.	
	% [*N*] (95% CI)	s.s.	% [*N*] (95% CI)	s.l.	% [*N*] (95% CI)	Status	% [*N*] (95% CI)	Status	% [*N*] (95% CI)	Status	% [*N*] (95% CI)	Status
0.05% Deltamethrin	**100** [487]	S	**99.5** [2241]	S	**100** [45]	S	**98.7** [1059]	S	**31.8** [346]	R	**100** [48]	S
	(99.2–100)		(99.2–99.8)		(92.1–100)		(97.8–99.3)		(26.9–37.0)		(92.6–100)	
0.05% Lambdacyhalothrin	**100** [186]	S	**99.2** [757]	S	NA	NA	**99.7** [387]	S	**32** [50]	R	**100** [14]	S^b^
	(98.0–100)		(98.5–99.8)				(98.6–100)		(19.5–46.7)		(76.8–100)	
0.05% Alphacypermethrin	**100** [101]	S	**100** [172]	S	NA	NA	**100** [62]	S	**64.3** [42]	R	NA	NA
	(96.4–100)		(97.9–100)				(94.2–100)		(48.0–78.5)			
0.75% Permethrin	**100** [99]	S	**100** [106]	S	NA	NA	**100** [88]	S	**10.7** [28]	R^b^	NA	NA
	(96.3–100)		(96.6–100)				(95.6–100)		(2.3–28.2)			
4% DDT	**100** [94]	S	**95.9** [1812]	PR	NA	NA	**78.5** [762]	R	**18.9** [127]	R	**89.5** [19]	R^b^
	(96.2–100)		(94.9–96.8)				(75.4–81.4)		(12.5–26.8)		(66.9–98.7)	
5% Malathion	**100** [97]	S	**100** [1051]	S	NA	NA	**97.2** [567]	PR	**99.5** [203]	S	**100** [51]	S
	(96.3–100)		(99.7–100)				(95.5–98.4)		(97.0–100.0)		(93.0–100)	
0.25% Pirimiphos-methyl	**100** [114]	S	**100** [287]	S	NA	NA	**99.2** [129]	S	**100** [39]	S^b^	NA	NA
	(96.8–100)		(98.7–100)				(95.8–100)		(91.0–100)			
0.1% Bendiocarb	**100** [96]	S	**99.9** [1177]	S	NA	NA	**98.5** [922]	S	**94** [133]	PS	**100** [45]	S
	(96.2–100)		(99.5–100)				(97.5–99.2)		(88.0–97.0)		(92.1–100)	

Results are shown as 24 h mortality in percent (bold) with 95% confidence intervals given (95% CI) along with the total number of mosquitoes exposed [*N*]. Susceptibility status based on mortality rates is classified according to the WHO criteria as follows: S (susceptible) denotes 98–100%, PR (possible resistant) denotes 90–97% and R (resistant) denotes < 90% mortality. Cells designated NA are indicative of no data available as no bioassays were conducted or data were omitted due to sample size being too low

^a^Insecticide susceptible *An. farauti* s.s. colony maintained at PNGIMR

^b^Inconclusive because of low number of mosquitoes tested

*Anopheles punctulatus* s.l. populations (Table 1) showed susceptibility to all tested insecticides at discriminating concentrations except for DDT, for which resistance was indicated, with 95.8% (95% CI 94.8–96.7%) 24 h mortality. Minority species of *Anopheles* spp. (mainly *An. bancroftii* and *An. longirostris*) numbers were too low to draw any conclusions for most insecticides except for deltamethrin, where susceptibility was indicated.

*Aedes aegypti* populations present in urban centres across PNG exhibited pyrethroid and DDT resistance as indicated by an average mortality of 31.8% (95% CI 26.9–37.0%) against deltamethrin, 32.0% (19.5–46.7%) for lambda-cyhalothrin and 18.9% (95% CI 12.5–26.8%%) for DDT (Table 1). Bendiocarb resistance was observed with 94.0% mortality (95% CI 88.0–97.0%). Resistance to other pyrethroid insecticides, alphacypermethrin and permethrin was inconclusively indicated because of low sample numbers. *Aedes aegypti* remained susceptible to 5% malathion with 99.5% (95% CI 97.0–100.0%) and pirimiphos-methyl with 100% mortality (95% CI 91.0–100.0%).

*Aedes aegypti* tested against deltamethrin and malathion in Morobe and NCD at the discriminating concentrations recommended for *Aedes* mosquitoes by WHO in 2016 [22] at 0.03% and 0.8%, respectively, showed deltamethrin and malathion resistance. However, as shown above, these vector populations had high mortality rates against the recommended discriminating concentration recommended for *Anopheles* mosquitoes. This malathion concentration is approximately six times higher than the interim recommended *Aedes* discriminating concentration (0.8% vs. 5%). According to the WHO intensity bioassay criteria [2], the species, when results were combined across PNG, shows a low level of resistance intensity.

*Aedes albopictus* showed resistance to DDT (78.5%, 95% CI 75.4–81.4%) and possible resistance to malathion (97.2%, 95% CI 95.5–98.4%). *Aedes albopictus* showed susceptibility to deltamethrin (98.7%, 95% CI 98–100%), lambdacyhalothrin (100%), permethrin (100%) and bendiocarb (99%, 95% CI 98–100%). Similar to *Ae. aegypti* populations in Morobe and NCD, *Ae. albopictus* was resistant to malathion (43%, 95% CI 36–50%) and possibly resistant to deltamethrin (97%, 95% CI 90–99%) at the 2016 WHO recommended discriminating concentrations for *Aedes* mosquitoes [22].

Other *Aedes* species (which are mainly *Ae. scutellaris* from the northern PNG) were present in numbers too low to draw definitive conclusions. However, results suggest phenotypic resistance against DDT with 24 h mortality of 89.5% (95% CI 66.9–98.7%).

### Spatial and species-specific results of *An. punctulatus* s.l.

**Fig. 3:**
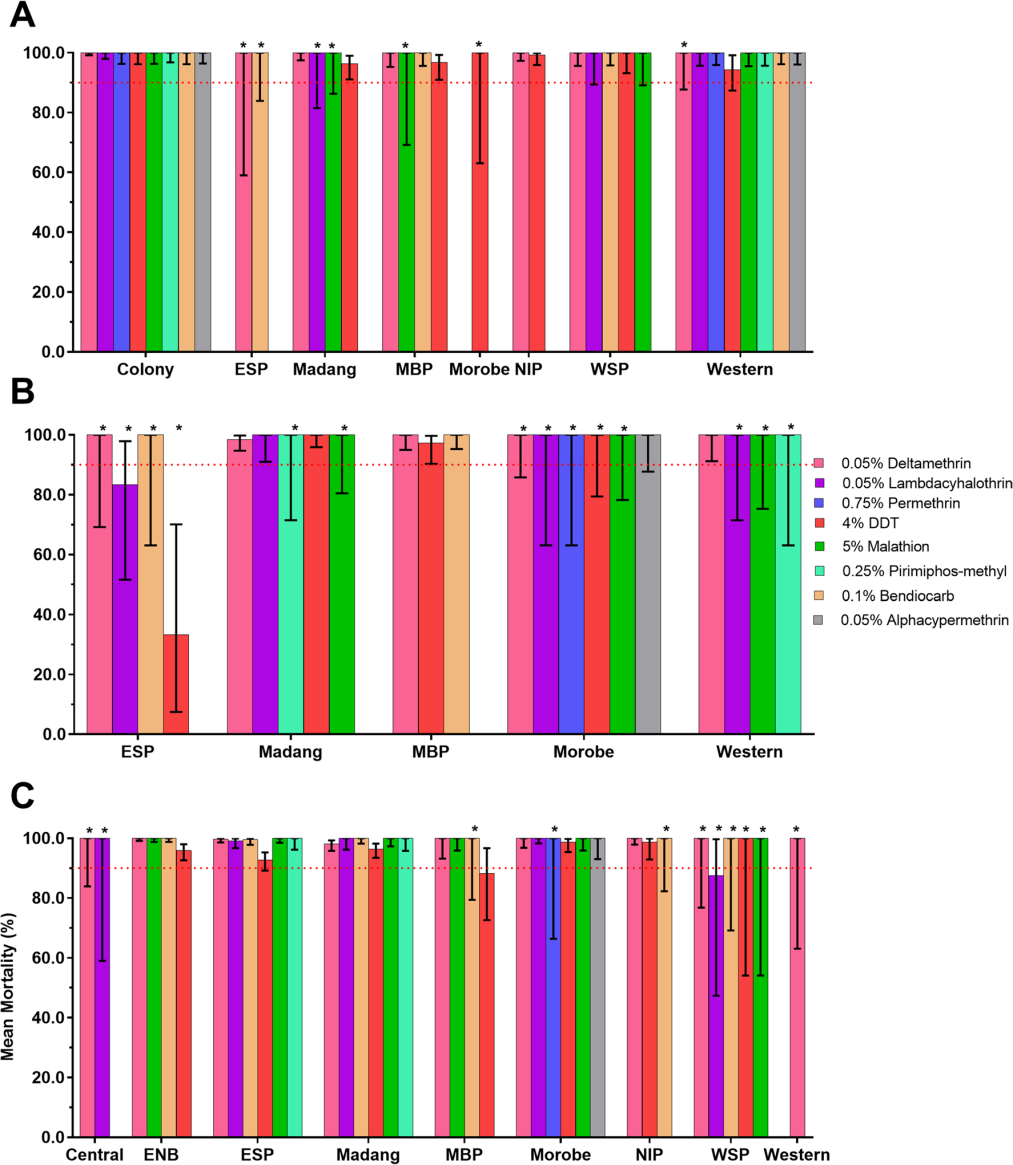
Mean mortality rates of the *Anopheles punctulatus* complex (*An. punctulatus* s.l.) for nine selected provinces of PNG against eight different insecticides. **A**: *Anopheles farauti*, **B**: *Anopheles koliensis*; **C**: *Anopheles punctulatus* s.s. Provinces surveyed indicated on the x-axis; abbreviations are: East New Britain (ENB), East Sepik (ESP), Milne Bay (MBP), New Ireland (NIP) and West Sepik (WSP); 95% CIs indicated by error bars. The WHO resistance threshold line for discriminating doses shown at 90% mean mortality. Sample numbers ≤ 5 not included and > 5 ≤ 30 indicated with asterisks. Controls not included

Species-specific 24 h mortality rates for the three primary *Anopheles* morphospecies *An. farauti*, *An. punctulatus* s.s. and *An. koliensis* in each province are shown in Fig. 3. When data were stratified by morphospecies and province, sample size usually became quite small, limiting the confidence in the conclusions that could be drawn from the analyses.

Data indicate susceptibility against all tested insecticides in all morphospecies and provinces, including deltamethrin. Results from the *An. koliensis* population in East Sepik province indicated higher than average levels of DDT and lambda-cyhalothrin resistance, with 24 h mortality of 33.3% (95% CI 7.49–70.1%) and 83.3% (95% CI 51.6–97.7%), respectively. Results from the *An. punctulatus* population also indicated higher than average levels of DDT resistance in Milne Bay province, with a mortality of 88.2% (95% CI 72.6–96.7%) and lambda-cyhalothrin resistance in West Sepik province, with a mortality of 87.5% (95% CI 47.4–99.7%).

DDT results also indicated possible resistance in the *An. farauti* population in Madang, Milne Bay and Western provinces with 24 h mortality rates ranging between 94% and 97%, and in the *An. punctulatus* population of East New Britain, East Sepik and Madang provinces, with 24 h mortality rates ranging between 92 and 97% (Fig. 3).

### Spatial and species-specific results for *Aedes aegypti* and *Ae. albopictus*

**Fig. 4:**
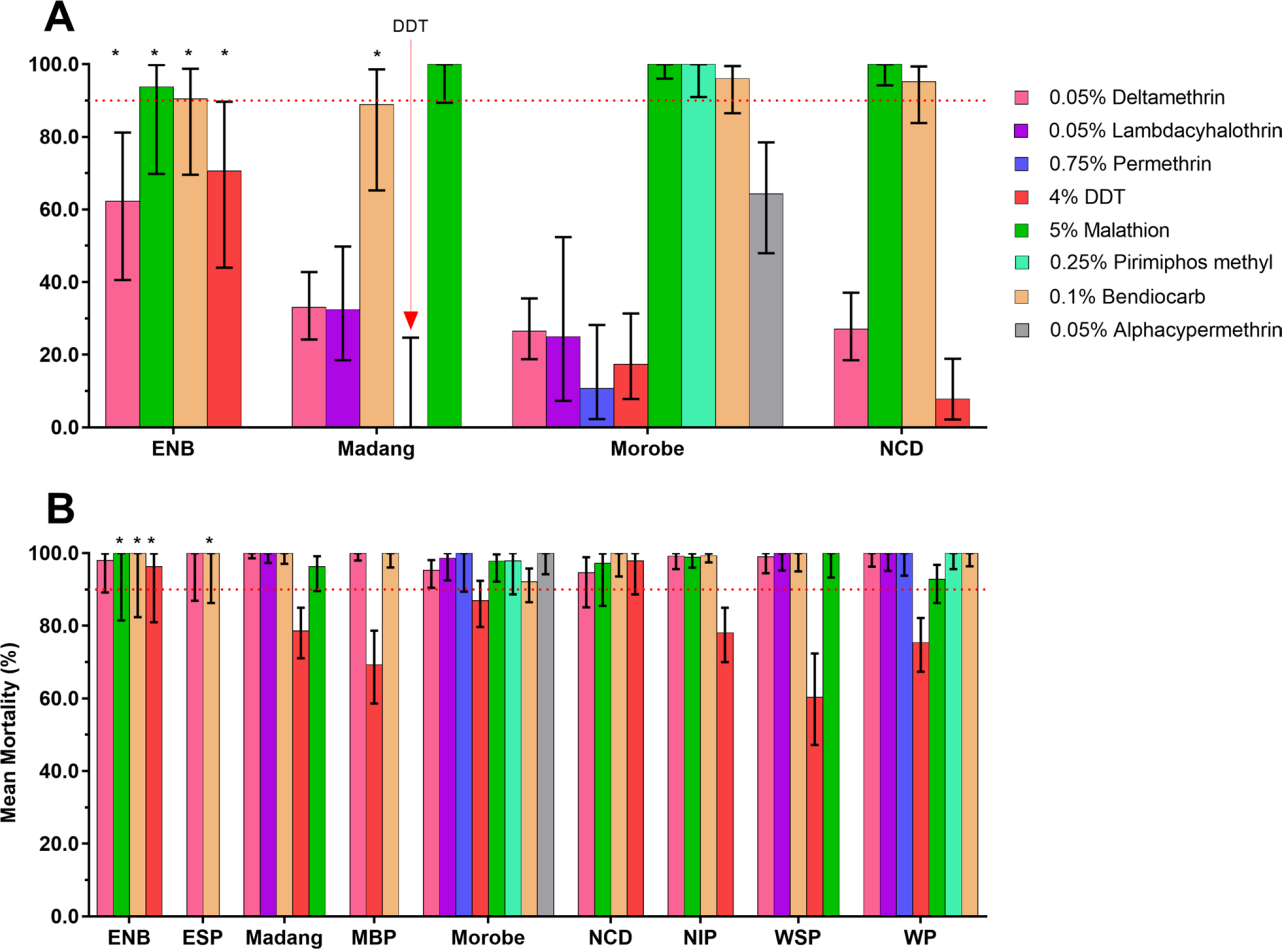
Mean mortality rates of populations of *Ae. aegypti* (**A**) and *Ae. albopictus* (**B**) across different provinces in PNG against different insecticides. Error bars are 95% confidence intervals. *Low sample size (< 30 mosquitoes); however, mortality rates are displayed to show the landscape of insecticide susceptibility across PNG

**Fig. 5:**
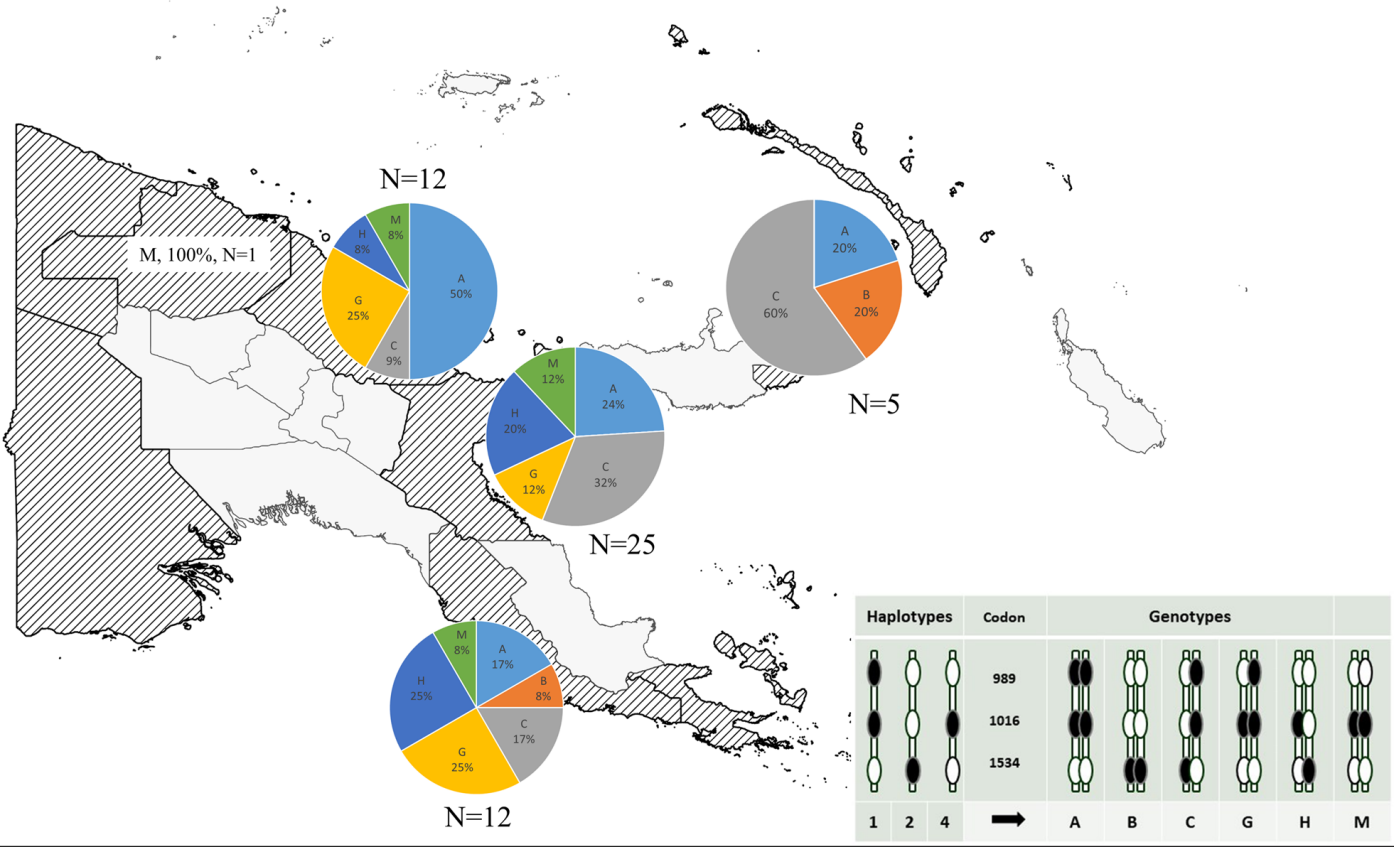
Distribution of *kdr* mutations in 55 *Aedes aegypti* samples from 5 provinces in PNG. The legend shows the haplotypes and resulting genotypes that were identified. Each genotype was assigned a letter. The size of the pie charts is arbitrary and not reflecting sample size. East Sepik Province only had one sample; therefore, no pie chart is presented

Species-specific 24 h mortality for the two primary *Aedes* species *Ae. aegypti* and *Ae. albopictus* in each province is shown in Figs. 4, 5.

*Aedes aegypti* were found in four population centres, namely Port Moresby (NCD), Lae (Morobe), Madang (Madang Province) and Kokopo and Rabaul (East New Britain Province). Resistance was observed against deltamethrin, lambda-cyhalothrin, DDT, malathion (5%) and bendiocarb (Fig. 4). Deltamethrin tested across all four *Ae. aegypti* populations showed 24 h mortality rates ranging from 17 to 63%. Lambda-cyhothrin resistance was observed in Madang with mortality rates of 32.4% (95% CI 18.5–49.8%) and in Morobe with 25% 24 h mortality (95% CI 7.3–52.4%). DDT resistance with mortality ranging from 0 to 71% was detected in all four provinces. Malathion (5%) susceptibility was observed in Morobe, NCD and Madang, but possible resistance was detected in East New Britain with 24 h mortality of 93.8% (95% CI 69.8%–99.8%). Possible resistance to bendiocarb with mortality ranging from 89 to 95% was observed in Morobe, NCD, ENB and Madang.

*Aedes albopictus* was found in all nine surveyed provinces and mainly showed susceptibility or possible resistance against the panel of insecticides tested except for DDT, against which confirmed resistance was indicated (Table 1). DDT resistance was observed with rates between 60 and 79% in West Sepik, Milne Bay, Western, Morobe, New Ireland and Madang. Possible DDT resistance was observed in East New Britain with 96.3% (95% CI 81.0–99.9%) mortality and in NCD with 97.9% (95% CI 88.7–100%) mortality. Possible deltamethrin resistance was observed in Morobe (95.3%, 95% CI 90.5–98.1%)) and in NCD [94.6% (95% CI 85.1–98.9)]. All *Ae. albopictus* in the seven provinces outside of Morobe and NCD were deltamethrin susceptible with mortality rates ranging from 98 to 100%. The species was fully susceptible to the other pyrethroid insecticide, lambda-cyhalothrin, in Madang, West Sepik and Western (100% mortality). The Western Province *Ae. albopictus* population was also fully susceptible to permethrin. Possible malathion (5%) resistance was observed in Morobe with mortality rates of 97.8% (95% CI 92.2–61%), NCD with 97.2% (95% CI 85.5–99.9%), Madang with 96.3% (95% CI 89.6–99.2%) and Western Province with 92.8% (95% CI 86.3–96.8%). *Aedes albopictus* in New Ireland and West Sepik were malathion susceptible with 24 h mortalities between 99 and 100%. Bendiocarb susceptibility was indicated with mortality rates ranging from 99 to 100% in all provinces except in Morobe province where possible resistance was indicated with 92.1% (95% CI 86.5–95.8) mortality. For pirimiphos-methyl susceptibility in *Ae. albopictus* was observed in Western Province [100% (95% CI 95.6–100.0%)] and possible resistance in Morobe, with 97.9 (95% CI 88.7–100%) mortality.

### Molecular analyses in *Ae. aegypti* and *Ae. albopictus*

Mutations in the *Vssc* gene in *Ae. aegypti* were found to be common at codons 1016, 1534 and 989 in the PNG sample. Six composite *Vssc* genotypes arising from four haplotypes were identified in the *n* = 55 samples of *Ae. aegypti* from five PNG provinces (Table 2). Three of the genotypes are known to confer pyrethroid resistance. No wildtype genotype or haplotype was identified in the sample.

**Table 2:** Six composite genotypes identified in *Aedes aegypti* in PNG

Codon				
1016	1534	989	*n*	Frequency
GG	TT	CC	15	0.273
TT	GG	TT	2	0.036
TG	TG	TC	14	0.255
GG	TT	TC	9	0.164
TG	TG	TT	9	0.164
GG	TT	TT	6	0.109

The most frequent genotype (frequency = 0.27) in the sample was a homozygous mutation at codon 1016 and 989 with the wildtype homozygote at codon 1534. Another common genotype found was the triple heterozygote at V1016G, F1534C and S989P (genotype frequency = 0.26). A new genotype (1016G in the homozygous state and not linked to 989P—now known as genotype M) for PNG was found at a frequency of 11% in the sample. The distribution of *Vssc* genotypes across the sampled provinces of PNG revealed resistance mutations to be widespread.

Genotyping by sequencing the region including codon 1534 of the *Vssc* in *Ae. albopictus* showed that most mosquitoes in the sample were wildtype for the resistance mutation. However, one homozygote and four heterozygotes for this mutation were found in a sample of 166 individuals. The mosquitoes carrying this mutation were collected in Milne Bay and Morobe Provinces (Table 3).

**Table 3:** Sequencing results for the region including codon 1534 of the *Vssc* in *Aedes albopictus*

Province	1534		*n*	Freq. in province
East New Britain	TT	WILDTYPE	26	1.00
East Sepik	TT	WILDTYPE	44	1.00
Milne Bay	TT	WILDTYPE	9	0.82
Milne Bay	TG	F1534C	1	0.09
Milne Bay	GG	1534C	1	0.09
Morobe	TT	WILDTYPE	20	0.91
Morobe	TG	F1534C	2	0.09
New Ireland	TT	WILDTYPE	12	1.00
West Sepik	TT	WILDTYPE	10	1.00
Western	TT	WILDTYPE	41	0.98
Western	TG	F1534C	1	0.02

Most samples were wildtype but low frequencies of resistance mutations were identified in Milne Bay, Morobe and Western Provinces

## Discussion

The present study provides the largest currently available dataset on IR status of malaria and arbovirus vectors in PNG. Using standard WHO tube bioassays, phenotypic resistance of *Anopheles* and *Aedes* species against important insecticides used in public health programmes in nine high-burden provinces was characterised.

The three primary malaria vector species, *An. farauti, An. punctulatus* s.s. and *An. koliensis*, have been previously profiled as susceptible to deltamethrin, lambda-cyhalothrin and DDT in Madang, East Sepik, Manus, Milne Bay and East New Britain provinces using similar methods [13, 14]. Susceptibility of *An. punctulatus *s.l. to the AIs used in malaria control in PNG, in particular deltamethrin, which has been the single AI used in LLINs in PNG for over 15 years, indicates sustained bioefficacy against these vector species. This is interesting as PNG has achieved > 80% LLIN coverage nationwide with > 50% usage rates since 2014 [28]. Vector behaviours leading to reduced contact with LLINs such as outdoor, early evening biting and opportunistic feeding behaviour could play a role in maintaining susceptibility to deltamethrin [29].

This study indicates that the *An. punctulatus* populations in East Sepik Province and East New Britain Province are showing signs of emerging phenotypic resistance to DDT and lambda-cyhalothrin. This is alarming, as widespread emergence of pyrethroid resistance in PNG would compromise current public health vector control strategies. Data also indicate DDT resistance in *An. farauti* and *An. koliensis*; however, this could not be conclusively shown because of the low numbers of the species tested in East Sepik. It is important to note that *An. koliensis* was shown to be resistant to DDT in neighbouring Irian Jaya, Indonesia [30]. It is imperative that future IR surveillance activities confirm the resistance status of these important malaria vector species. Whether the observed signs of DDT resistance in Anopheles populations in some areas are a remnant of historic DDT usage in PNG [31], agricultural usage of DDT and/or pyrethroids [32] or emerging cross-resistance with pyrethroids [33] is yet to be elucidated. For example, the observed resistance against lambda-cyhalothrin as well as DDT in some provinces could be suggestive of cross-resistance between DDT and lambda-cyhalothrin.

Furthermore, it is unclear whether the cross-resistance profile (DDT-lambda-cyhalothrin) observed in the PNG vector species is driven by target site mutations or through a metabolic detoxification pathway such as that observed in the South American malaria vector, *Anopheles darlingi* [34]. Thus, there is an urgent need to confirm the underlying resistance mechanisms using molecular analysis tools such as *Vssc* genotyping and population genomic tools such as whole-genome sequencing and investigation of gene copy number variation [35]. Additional biochemical and biological assays, including synergist bioassays, are also needed to investigate whether metabolic resistance is present [36].

*Aedes aegypti* and *Ae. albopictus* are the two common arboviral vectors in PNG. Previous profiling of these species in Madang and Port Moresby (NCD) reported pyrethroid resistance in *Ae. aegypti* and DDT resistance in *Ae. albopictus* in Madang. While both species in the two areas were susceptible to malathion, *Ae. aegypti* also showed resistance to DDT and bendiocarb (not fully confirmed due to low sample size) and *Ae. albopictus* was susceptible to lambda-cyhalothrin [15].

The present study confirms the presence of resistance to deltamethrin, lambda-cyhalothrin, DDT and malathion in *Ae. aegypti* and *Ae. albopictus*, in several populations, highlighting a critical need to understand the landscape of insecticide use for pest control (private, public health, agricultural and industrial sectors) in PNG. DDT was last used almost 50 years ago in the national malaria control programme [37]; however, illegal use of DDT has been reported in PNG for either public health or agriculture [32]. Products containing a number of AIs including malathion and lambda-cyhalothrin are frequently used in PNG [32] suggesting that selective pressure from these AIs could be contributing to the resistance observed in *Ae. aegypti* and the emergence of resistance in *Ae. albopictus*.

The capital cities of Port Moresby (NCD) and Lae (Morobe) are the most urbanized and densely populated areas in PNG. Both *Aedes* species thrive in these environments, similar to elsewhere in Asia [38], Latin America [39] and Africa [10], posing a high risk of arboviral transmission, in particular dengue fever, chikungunya and Zika. The observed widespread resistance in *Ae. aegypti* and *Ae. albopictus* to multiple insecticides in these provinces (Morobe and NCD) highlights a critical need for a harmonized vector control plan that considers these resistance profiles. Furthermore, building capacity for routine mosquito surveillance in PNG and strengthening partnerships across PNG research institutions, government departments and health laboratories is critical to reduce these important mosquito vector populations and thus reduce disease transmission [40].

The pyrethroid resistance within the *Ae. aegypti* populations in PNG is conferred, at least in part, by three or more different *kdr* genotypes. The most frequent genotype, a homozygous mutation at codon 1016 and 989 with the wildtype homozygote at codon 1534, is a common genotype also found in Bali and other locations throughout Southeast Asia and the Pacific, and it confers a high level of resistance to Type I and II pyrethroids [24, 41]. The heterozygote at V1016G, F1534C and S989P is found commonly in *Ae. aegypti* in other countries in the region and confers a low to moderate level of resistance to Type I and II pyrethroids [42]. The homozygote 1534C occurring alone is rare in the PNG sample, but confers a low level resistance to Type I pyrethroids [42]. A new genotype for PNG (1016G in the homozygous state and not linked to 989P—now known as genotype M) has not been found in recent studies of *Ae. aegypti* in the Indo-Pacific region [24], but does exist in Taiwan, and its association with resistance has not been ascertained [43]. The two remaining genotypes identified in this study contain the haplotype that makes up genotype M and their association with pyrethroid resistance is yet to be tested. The role of metabolic resistance in the PNG mosquito populations has not been investigated. For both *Aedes* species studied in PNG, it will be important to determine whether the *Vssc* resistance mutations have arisen because of local selection or have arrived with invasive mosquitoes as has been shown in other parts of the Indo-Pacific region [24]. Whatever their origin, the continued presence of the *Vssc* mutations implies that they are being maintained because of selection with pyrethroid insecticides in PNG as there are known fitness costs associated at least with the 1016G/989P mutations [44, 45].

PNG has so far avoided pyrethroid resistance in anopheline vectors. It is important to aim to maintain pyrethroid susceptibility in PNG vectors by promoting sustainable and responsible use of insecticides in both public health and agriculture sectors in PNG. The WHO-recommended IR management guidelines for malaria vectors suggest strategies such as rotation of insecticides and combination of interventions such as LLINs and IRS to delay resistance development [46]. In addition, increased capacity-building and better resourcing of IR monitoring efforts in PNG are critical. A national strategic plan for vector surveillance and control as well as strong leadership by the National Department of Health is required. Facilitation and coordination of sub-national partnerships between public health and private sector (e.g., agricultural) stakeholders is critical for effective IR management in PNG [40].

## Conclusion

This study shows that *An. punctulatus,* an important malaria vector species in PNG is resistant to DDT, with possible cross-resistance to the pyrethroid insecticide lambda-cyhalothrin. Monitoring of the IR status of malaria vector species needs to be maintained. Non-pyrethroid-based solutions for malaria vector control such as next-generation LLINs should be piloted in PNG. There is currently no control programme targeting arbovirus vectors in PNG. As such, control of arbovirus vector species such as *Ae. aegypti* in PNG needs to be strengthened at the national and provincial levels. The existing toolbox for controlling these vectors is already compromised because of presence of pyrethroid, organochloride and organophosphate resistance. Continued monitoring of all of the important vector species in PNG using both phenotypic and molecular assays is crucial to identify and develop effective strategies to mitigate the spread of resistance.

## Data Availability

Data used in this present study are presented within the article.

## Abbreviations

IR: Insecticide resistance
LLINs: Long-lasting insecticidal nets
PNG: Papua New Guinea
WHO: World Health Organization
DDT: Dichlorodiphenyltrichloroethane
*Vssc*: Voltage-sensitive sodium channel
